# Risk, risk factors, and screening of malignancies in dermatomyositis: current status and future perspectives

**DOI:** 10.3389/fonc.2025.1503140

**Published:** 2025-06-04

**Authors:** Shubei Liu, Zhihong Zhang, Shushan Yan, Chunjuan Yang, Bin Wang, Minning Shen, Zhenhua Wang, Donghua Xu

**Affiliations:** ^1^ Department of Rheumatology and Immunology, Weifang People’s Hospital, Shandong Second Medical University, Weifang, China; ^2^ Department of Rheumatology and Immunology, Nanjing First Hospital, Nanjing Medical University, Nanjing, China; ^3^ Cultural Propaganda Office, Weifang People’s Hospital, Shandong Second Medical University, Weifang, China; ^4^ Department of Gastrointestinal and Anal Diseases Surgery, The Affiliated Hospital of Shandong Second Medical University, Weifang, China; ^5^ Medical Research Center, Weifang People’s Hospital, Shandong Second Medical University, Weifang, China; ^6^ Department of Dermatology, Weifang People’s Hospital, Shandong Second Medical University, Weifang, China

**Keywords:** dermatomyositis, cancer, risk factors, screening, risk assessment

## Abstract

Dermatomyositis (DM) is an idiopathic inflammatory myopathy with characteristic cutaneous inflammation and heterogeneous systemic involvements, and is strongly associated with risk of malignancy. This review summarizes the incidence of malignancies, risk factors associated with malignancies, and cancer screening methods in DM patients. Large population-based cohort studies and meta-analyses have provided strong evidence for the significantly elevated incidence of malignancies in DM patients. Common malignancies occurring in DM patients mainly include ovarian cancer, lung cancer, breast cancer, pancreatic cancer, stomach cancer, hematologic malignancies, and colorectal cancer. Clinicians should cautiously consider the risk of malignancy in DM patients during diagnosis and treatment, conducting regular screening and monitoring to facilitate early detection and treatment of malignancies. Among myositis-specific antibodies, anti-transcription intermediary factor 1γ antibodies are strongly linked to malignancy risk. Other factors such as older age, male gender, dysphagia, skin necrosis, cutaneous vasculitis, rapid onset of the disease, elevated creatinine kinase, and elevated C-reactive protein are closely associated with the risk of malignancy. DM patients with these features need receive screening for malignant tumors or close monitoring and follow-up. DM patients, especially those within 3 years of onset, have a high risk of cancer and should receive careful cancer screening according to their risk stratification. Conventional screening tools such as imaging examinations and tumor marker tests are not effective in detecting malignancies among DM patients. Current cancer screening workflows available for DM patients largely mirror those used in the general population but may not fully address DM-specific characteristics, and the best strategy for screening cancer in DM patients is still lacking. To facilitate earlier detection and diagnosis of DM-associated cancer and thereby improve outcomes, more effective cancer detection tools and personalized malignancy screening workflows specifically tailored to the features of DM and their individual risk stratification are warranted.

## 1 Introduction

Idiopathic inflammatory myopathies (IIM) are a heterogeneous group of autoimmune diseases typically involving skeletal muscles and/or affecting other systems or organs, such as the skin, lungs, and joints, collectively known as myositis (1). Until recently, the complex etiology and incompletely understood pathophysiology of IIM have posed challenges for both precise diagnosis and effective treatment (1). Multiple factors, such as T cells, neutrophils, and monocytes, are implicated in the pathogenic mechanisms of IIM (1). The incidence of IIM is estimated to be about 0.2 to 2 per 100,000 person-years, and the prevalence is estimated to be 2 to 25 per 100,000 people (2–4). Based on autoantibody evaluation, IIM can be classified into dermatomyositis (DM), polymyositis (PM), anti-synthetase syndrome, immune-mediated necrotizing myopathy, inclusion body myositis (IBM), and overlap myositis (5, 6). Among these diseases, DM is characterized by specific cutaneous and muscular findings and heterogeneous systemic involvements (7). Organ damage and comorbidities are common in most IIM patients, resulting in a high disease burden and poor quality of life (8). Therapeutic options for IIM patients mainly include glucocorticoids and immunosuppressive agents, but effectively controlling disease severity remains challenging, necessitating the development of more effective treatment options for IIM (1).

IIM including DM is associated with an increased cancer risk especially before and after 3 years of onset, and malignancy is one of the most serious risk factors for death in patients with IIM (9, 10). Among IIMs, DM is the most documented form associated with cancer risk, and numerous clinical observational studies have revealed a significantly increased risk of malignancy in DM patients (11–13). Some scholars propose that DM may be a paraneoplastic disorder in some patients with cancers, but the mechanism involved in the development of DM in cancer patients is unclear (11, 14, 15). Malignancy can be diagnosed before, concurrent with, or after the diagnosis of IIM, with the highest risk occurring during the year prior to and the year after IIM diagnosis (11–13). Therefore, regular screening and monitoring to facilitate early detection and treatment of malignancies are necessary in DM patients especially with risk factors.

Myositis associated with malignancies is often referred to as cancer-associated myositis (CAM), and it is defined as patients who develop tumors within 3 years before or after the diagnosis of myositis (11, 16, 17). CAM occurs in about 10.0%-15.0% of IIM patients, and its incidence is increasing in recent years (18–21). The increasing use of immune checkpoint inhibitors (ICIs) in cancer patients also result in an increasing occurrence of myositis in cancer patients (22, 23). Owing to the coexistence of the increased cancer risk associated with immunosuppressive therapy for IIM and the increased myositis risk caused by ICIs for cancer, the occurrence of CAM in either myositis patients or cancer patients can cause a great challenge to the adequate treatment choice between two diseases (17, 24). CAM is a serious complication for IIM patients, and it also correlate with deleterious outcomes such as ICIs withdraw and cancer progression in cancer patients (25–27). For instance, a study by Allenbach et al. found that ICIs-induced myositis was the earliest and most lethal complication among rheumatic and musculoskeletal toxicities in cancer patients receiving ICIs treatment (28).

Early detection of potential malignancies is crucial for improving survival rates and quality of life in cancer patients, and it is also true among DM patients (29, 30). Given the high risk of cancer in DM patients especially those within 3 years of onset, assessing malignancy risk and cancer screening methods are key focuses in DM patients (31–33). Risk factors for malignancies in DM patients have been explored by numerous studies, which are important to risk stratification and subsequent cancer screening in DM patients (16, 34). For instance, some myositis-specific antibodies such as anti-transcription intermediary factor 1γ (anti-TIF1γ) antibody are risk factors related to cancer among DM patients (35). DM can be classified into several types, with the most common being adult dermatomyositis (ADM) and juvenile dermatomyositis (JDM). The occurrence of JDM is associated with various risk factors; however, unlike ADM, JDM is rarely linked to malignancies (36). The objective of this review is aimed to systematically summarize the incidence of malignancies, risk factors associated with malignancies, and cancer screening methods in DM patients by pooling all relevant studies published up to date. Future perspectives on the screening of DM-associated cancers are also discussed to facilitate earlier detection and diagnosis of DM-associated cancer, thereby improving DM outcomes. This review also provides insight into the exploration of more effective cancer detection tools and personalized malignancy screening workflows of DM patients.

## 2 Cancer risk among DM patients

The unusually high occurrence of malignancies in DM patients has attracted scholars’ attention since the 1950s, and the increased risk of malignancies in DM patients has been proven in numerous studies over the past 60 years (12, 13, 15, 37–40). Epidemiological studies and meta-analyses examining the relationship between DM and malignancy risk are summarized in the following sections, which have provided strong evidence of the significantly elevated incidence of malignancies in DM patients (**Table 1**) and underscored the importance of tumor screening in this population.

**Table 1:** Cancer risk among DM patients.

Study	Country of origens	No. of DM	Mean age or age range	Sex (M /F)	Cancer incidence	Cancer patient sex (M/F)	Cancers	Time of cancer diagnosed after DM
Curtis AC et al. (37)	USA	45	2-69y	17/28	17%	1/7	hodgkin’s disease, scirrhous carcinoma of breast, squamous cell carcinoma of cervix, adenocarcinoma of breast, etc.	18.6 mo.
Arundell FD et al. (38)	USA	35	3-76y	25/10	34.3%	3/9	prostate cancer, vocal cord cancer, bronchogenic cancer, breast cancer, lymphomas, ethmoid sinus cancer, rectum cancer, etc.	6 mo.
Sigurgeirsson B et al. (42)	Sweden	392	11 mo.-88y	145 /247	15.6%	25/36	gastric cancer, colorectal cancer, pancreatic cancer, lung cancer, breast cancer, ovarian cancer, renal cancer	1 year and 5 years of DM diagnosis
Chow WH et al. (15)	Denmark	203	48.8y	NA	SIR=3.8	NA	buccal and pharynx cancer, digestive system cancer, lung cancer, breast cancer, ovarian cancer, prostate cancer, lymphatic and hematopoietic system cancers	1 year of DM diagnosis
Yosipovitch G et al. (60)	USA and Singapore	34	48y	10/24	20%	NA	nasopharyngeal carcinoma, colon cancer, thymic carcinoma	NA/NA
Hill CL et al. (40)	Sweden, Denmark, and Finland	618	M:55.6y F:55.4y	NA	SIR=3.0	23/64	ovarian, lung, pancreatic, stomach, and colorectal cancers, and for lymphomas, etc.	1 year of DM diagnosis
Buchbinder R et al. (45)	Australia	85	51.7y	30/55	42.4%	NA	lung, ovary, colorectal, cervix, stomach, breast, prostate and uterus cancer, etc.	3 year of DM diagnosis
Stockton D et al. (46)	Britain	286	NA	97 /189	SIR=7.7	20/30	carcinoma of nesophagus, stomach, colon, lung, breast, cervix uteri, ovary,etc.	3 months after DM diagnosis
Chen YJ et al. (57)	China	91	47.5y	40/51	18%	NA	nasopharyngeal cancer, lung cancer, liver cancer, gastric adenocarcinoma, low-grade lymphoma, breast adenocarcinoma, uterine cervix carcinoma, etc.	NA
Zhang W et al. (58)	China	678	NA	255 /423	17%	63/52	nasopharyngeal cancer, lung cancer, liver cancer, ovarian cancer, cholangial cancer, esophageal cancer, colon cancer, etc.	1 year of DM diagnosis
Huang YL et al. (59)	China	1059	42.4y	329 /730	12.8%	50/86	nasopharyngeal cancer, lung cancer, liver cancer, breast cancer, etc.	1.14y
Sung YK et al. (61)	Korea	526	48.8y	167 /359	19.6%	NA	lung, hematologic and liver malignancy, thyroid and oropharynx malignancy, prostate, stomach, breast, and thyroid malignancy	1 year of DM diagnosis
Chen YJ et al. (62)	China	1012	41.8y	315 /697	9.4%	39/56	nasopharyngeal cancer, lung/mediastinum cancers, bone/joint cancers and lymphomas/leukemias, etc.	1 year of DM diagnosis
Chen D et al. (63)	China	246	49.2y	107 /139	24.4%	41/19	nasopharyngeal carcinoma, ovarian cancer, lung cancer, colon carcinoma, thyroid carcinoma, lymphoma, breast carcinoma, etc.	11.4 mo.
Hsu JL et al. (64)	China	1100	49.1y	395 /705	5.55%	28/33	nasopharyngeal cancer, breast cancer, liver and intrahepatic bile ducts cancer, bronchus and lung cancer, etc.	1 year of DM diagnosis
Azuma K et al. (66)	Japan	70	51y	21/49	24%	NA	gastric cancer, colon cancer, ovarian cancer, breast cancer, etc.	NA
Chang L et al. (67)	China	202	NA	109 /93	8.83%	35/30	lung, thyroid, breast and cervical cancer, esophageal cancer, thyroid cancer, breast cancer, nasopharyngeal carcinoma, etc.	1 year of DM diagnosis
Antiochos BB et al. (68)	USA	61	56.7y	15/46	39.3%	8/16	breast, lung, pancreas, and colon cancer, etc.	1 year of DM diagnosis
Leatham H et al. (69)	USA	400	51.9y	77 /323	15.8%	NA	breast, hematologic, colorectal, and prostate cancer, etc.	1 year of DM diagnosis
Bowerman K et al. (70)	USA	201	51y	26 /175	11.6%	NA	breast, ovarian, lung, hematopoietic cancer, etc.	1 year of DM diagnosis
Tripathi R et al. (71)	USA	39253	NA	10564/28689	10.9%	3042 /1236	bronchial, non-Hodgkin’s lymphoma, head/neck, bladder, esophageal, kidney, stomach, breast and ovarian cancer, etc.	NA

NA, not available; M, male; F, female; A, age; T, time; DM, Dermatomyositis; SIR, Standardized Incidence Ratio.

### 2.1 Increased risk of malignancies in DM patients

Numerous studies have been published to evaluate the risk of malignancies in DM patients (12, 15, 37–39, 41, 42). A study by Curtis et al. found that 17.7% of 45 DM patients had some forms of malignant diseases, with the majority being female (37). DM preceded the appearance of malignant diseases by an average of 18.6 months, and DM severity was improved following treatment of malignant diseases in most patients (37). Another study by Arundell et al. reported that the incidence of malignancies in DM patients over the age of 40 years was more than 50%, which was markedly higher than the expected incidence of cancer for the general population (38). Improvement in DM severity occurred after treatment of cancers in six of nine patients, but exacerbation of DM after initial improvement occurred with the progression of malignancies in half of these six patients (38). Sigurgeirsson et al. conducted a population-based cohort study of 392 DM patients and found that the relative risk of cancer was 2.4 in male patients and 3.4 in female patients compared with the general population (42). A nationwide cohort study in Denmark showed that the overall cancer risk was significantly elevated among patients with DM (Standardized incidence ratio [SIR] = 3.8) (15). The cancer risk was increased approximately six-fold (SIR = 5.9) during the first year after the initial diagnosis of DM but declined steadily with increasing years since the initial diagnosis of DM (15). In a pooled analysis of 618 DM cases, DM was strongly associated with malignant diseases (SIR = 3.0), with the highest risk observed at the time of DM diagnosis (40). In brief, findings from numerous studies suggest that DM patients exhibit a significantly higher risk of malignancy compared to healthy controls or non-DM cohorts.

However, not all studies confirm this association, with some showing no significantly increased risk of malignancy in DM patients (41). The lack of significant findings may be due to selection bias in participants or methodological limitations in sample size. A meta-analysis of 10 cohort studies showed that DM patients are at a significantly increased risk for developing cancer compared with the general population, and the pooled SIR for the incidence of overall cancer in DM patients was 4.79 (43). Another meta-analysis of 20 studies showed that the pooled rate ratio (RR) of overall malignancy in DM patients was 5.50 compared with the general population (44). Therefore, findings from meta-analyses also provide strong evidence for the significantly elevated incidence of malignancies in DM patients.

### 2.2 Time-dependent pattern in cancer risk of DM patients

An obvious time-dependent pattern with the highest excess risk occurring around the time of DM diagnosis exists in the cancer risk of DM patients, suggesting a close temporal association between malignancy and autoimmune attack in DM patients (45–47). For instance, a population-based cohort study from Australia showed that the excess risk for malignancies in DM patients decreased over time following DM diagnosis, with SIRs of 4.4 in the first year, 3.4 between 1 and 3 years, 2.2 between 3 and 5 years, and 1.6 beyond 5 years (45). Manchul et al. found a higher frequency of malignancies in DM/PM patients compared to rheumatic disease controls and/or noninflammatory musculoskeletal controls, but observed no increase in the incidence rate of subsequent malignancies during follow-up (48). Another study revealed that cancer risk in DM patients could remain higher than healthy people in 5 years after diagnosis (40). Besides, a nationwide study in Sweden indicated that the types of cancers occurring before IIM diagnosis differed from those occurring after diagnosis (49). Before diagnosis, the malignancies with excess risk in IIM patients mainly included colorectal cancer, lung cancer, and ovarian cancer (49). After diagnosis, the malignancies with excess risk in IIM patients mainly included oropharyngeal cancer, cervical cancer, and skin cancer (49).

The close time-dependent correlation between myositis and cancer onset, and the phenomenon of myositis remission after tumor treatment, indicate that some myositis occurring in cancer patients may be a paraneoplastic myositis syndrome (PMS) (11, 14, 15, 50). CAM is commonly defined as those myositis patients who develop tumors within 3 years before or after the diagnosis of myositis (11, 14, 15, 50). Some myositis-specific autoantibodies (MSAs) are closely related to the increased risk of malignant tumors, and this group of antibodies is sometimes referred to as cancer-associated autoantibodies (CAAs) (35, 51). The most intensively studied CAAs are anti-TIF1-γ antibodies (35). Published showed that antibodies against TIF1-γ could present in more than 50% of patients with cancer-associated myositis (52).

### 2.3 Cancer type-specific risk in DM patients

Though there is no uniformity in the types of malignancies found in DM patients, several common types exist such as lung cancer, ovarian cancer, and breast cancer (15, 53). A population-based study of 618 DM patients demonstrated a strong association between DM and specific types of cancer, such as ovarian cancer, lung cancer, pancreatic cancer, stomach cancer, non-Hodgkin lymphoma, and colorectal cancer (40). In another Scottish population-based cohort study, significantly elevated risks were observed for lung cancer, cervical cancer, and ovarian cancer in patients with DM (46). However, elevated risks for some types of cancers were not statistically significant. For instance, a meta-analysis of 20 publications showed a significant association between DM and most site-specific malignancies, but no association with stomach and prostate cancers (44). Such a weak or nonsignificant association could be due to the low occurrence of those types of cancer or low statistical power caused by smaller sample sizes. Therefore, further large-scale, multicenter studies are needed to validate whether DM patients indeed have an increased risk of those types of cancers.

There are significant variations between different studies regarding the degree of cancer risk in DM patients (40, 44, 46). Obvious differences also exist in the incidence rates and the extent of elevated risks across different cancer types (54). Several possible explanations exist for these differences. Firstly, variations in the pathophysiology of these cancers may result in differing incidence rates in DM patients. Secondly, differences in geographical regions and populations may also influence the consistency in the degree of cancer risk. Thirdly, differences in study methodologies, such as the definition of malignancy-associated myositis, may lead to varying results. Finally, some studies may suffer from sample selection bias or low sample sizes, affecting the reliability of results.

### 2.4 Geographical differences in cancer risk of DM patients

The incidence risk and distribution of malignancies are influenced by genetic background and ethnicity, and there are obvious geographical differences in the incidence risk of some types of cancers, such as nasopharyngeal carcinoma (55, 56). Accordingly, different populations from various geographical areas may be at risk for different myositis-associated malignancies.

Epidemiological studies from Asian countries often report more cases of nasopharyngeal carcinoma and liver cancer in DM patients compared to studies from Europe and North America (57–61). In some Asian studies, nasopharyngeal carcinoma was the most common cancer in DM patients (57–60, 62–64). A systematic review of 14 case-series studies in Asian populations revealed that nasopharyngeal carcinoma was the most common malignancy in DM patients, and the types of malignancies associated with DM in Asians differed from those in Caucasians (65). Other common cancers in DM patients from Asian countries include lung cancer, breast cancer, ovarian cancer, colorectal cancer, gastric cancer, esophageal carcinoma, and hepatobiliary cancer (59, 61, 63, 65–67). However, among studies from Europe and North America, the most common cancers in DM patients include ovarian cancer, breast cancer, lung cancer, pancreatic cancer, hematologic malignancies, and colorectal cancer (20, 68–71).

Therefore, DM patients from various geographical areas have obvious differences in the incidence risks across some types of cancers. This geographical difference in cancer risk among DM patients indicate that ethnic background is a key factor for clinicians to consider while screening for cancers in DM patients.

### 2.5 Mechanisms involved in the development of CAM

Myositis patients who develop tumors within 3 years before or after myositis onset are often referred to CAM, which is a hallmark in terms of the association between cancer and myositis (17, 72, 73). The mechanism involved in the development of CAM is unclear, but it may be related to factors such as autoimmunity, genetic factors, and immune responses (74–78). The abnormal immune-inflammatory responses in DM could create a conducive environment for malignancy development; conversely, malignancies themselves could potentially trigger or exacerbate the autoimmune attaches in DM (74, 77). Some scholars believe that in DM patients with malignant tumors, the tumor is very likely to affect the immune system, leading to abnormal immune responses and increasing the risk of autoimmunity (11, 79, 80). Zampieri et al. proposed that tumor and/or tumor-derived factors in a particular subset of individuals genetically predisposed to autoimmunity may trigger autoimmune myopathic changes and the development of CAM, which is a possible molecular mechanism (81).

A possible hypothesis underlying CAM is that antibodies against myositis autoantigens begin as part of an immune response directed against cryptic epitopes in cancer tissues, resulting from a breakdown of immune tolerance caused by cross-reactivity and/or epitope spreading (74, 77). Tumor antigen expressions caused by genetic mutations and/or overexpression in cancer tissues may become myositis autoantigens and trigger an antitumor immune response together with an autoimmune response against muscles and other target tissues with similar epitopes (11, 79, 80). These autoantigens contain cryptic epitopes not previously encountered by the immune system, and may initiate both antitumor responses and autoimmune responses against muscles and other target tissues (74, 77). For instance, a study by Pinal-Fernandez et al. found that tumor tissues from anti-TIF1γ-positive CAM patients showed increased genetic alterations such as somatic mutations and loss of heterozygosity (LOH) in TIF1 genes, and higher expression of TIF1γ compared to anti-TIF1γ-negative myositis patients, suggesting a role of TIF1γ in the pathophysiology of CAM (82). TIF-1γ (TRIM33) belongs to the larger tripartite motif (TRIM) protein family which play key roles in important biological processes including cell proliferation, apoptosis, and immune responses (74, 83). Some members of TRIM protein family are important regulators of carcinogenesis, and are involved in the development and progression of malignancies (84). There are four members in the TIF-1 protein family, including TIF-1α (TRIM24), TIF-1β (TRIM28), TIF-1γ (TRIM33), and TIF-1δ. TIF-1γ can promote the cancer growth and metastasis through several signaling pathways, but it has been reported to act as a cancer disincentive factor rather than a promoter in certain types of cancer such as non-small-cell lung cancer and breast cancer (85). A study by Cordel et al. found 14 probable somatic variants in the TIF-1γ gene among four tumors from patients with anti-TIF1γ-positive CAM (86). Another study by Motegi et al. confirmed that TIF1γ was highly expressed in tumors (87). Autoantibodies against TIF-1γ, genetic alterations, and aberrant expression may disrupt the normal functions of TIF-1γ in biological processes such as cell proliferation, apoptosis, and immune responses, potentially promoting the development of CAM (11, 74, 85). However, the molecular mechanisms underlying CAM are still largely unknown and need to be explored in future research.

Myositis induced by ICIs is another example which proves the key role of abnormal immune-inflammatory responses in CAM (22, 88, 89). ICIs can augment anti-tumor immunity but also increase immune system activation in cancer patients, which can result in the development of potentially life-threatening immune-related adverse events (irAEs) including myositis (90, 91). A study by Guerra et al. found that all DM cases induced by ICIs were positive at high titers for anti-TIF1γ autoantibodies, proving an involvement of autoimmune responses in myositis induced by ICIs (92). Another study by Pinal-Fernandez et al. performed transcriptomic analyses of 200 muscle biopsies from patients with ICIs-induced myositis and patients with IIM including DM, and found that patients with ICIs-induced DM were like DM patients in terms of both anti-TIF1γ autoantibodies and overexpressed type 1 interferon-inducible genes (93). With the increasing use of ICIs in oncologic therapy, drug-induced DM is becoming more common and need be diagnosed and treated promptly (89, 94).

## 3 Risk factors of malignancies among DM patients

Identifying clinical risk factors for malignancy in DM patients helps stratify their management, enabling intensive cancer screening for high-risk individuals while considering minimal or no screening for low-risk ones (34, 95). Cancer risk stratification is helpful for improving the benefits of cancer screening in DM patients, and risk factors of developing malignancies should be evaluated in DM patients for this purpose (95, 96). A better understanding of malignancy risk factors among DM patients can help clinicians identify those at higher risk of cancer, thereby facilitating appropriate cancer surveillance at DM onset and during follow-up (34). Therefore, studies focusing on risk factors for malignancies among DM patients are crucial for effective cancer risk stratification. Numerous studies have explored potential risk factors for malignancies among DM patients, and their findings are summarized below (**Table 2**).

**Table 2:** Risk factors of malignancies among DM patients.

Clinical features	Symptoms and signs	Laboratory parameters	Autoantibodies	Serum cancer markers	Positive- or negative-related (P/N)	Ref.
Absence of ILD; DM complicated by diabetes mellitus	Dysphagia	/	/	/	N	(10)
	Arthritis or arthralgia; Fever; Raynaud phenomenon				P	
DM subtype		/	/	/	N	(19, 20)
	Raynaud phenomenon				P	(20)
/	/	/	Anti-TIF1γ antibodies and Anti-NXP2 antibodies positivity	/	N	(52)
/	/	/	/	/	N	(58, 71)
Absence of ILD	Dysphagia	Elevated ESR	/	/	N	(63)
Absence of ILD	Dysphagia	/	/	/	N	(66)
Absence of ILD	Dysphagia	Decreased Albumin	/	/	N	(67)
	Arthritis or arthralgia; Fever				P	
/	Cutaneous necrosis or ulceration		/	/	N	(80)
	Arthritis or arthralgia; Fever; Raynaud phenomenon		ANA positivity		P	
Refractory or drug-resistant DM	/	/		/	N	(95)
			Anti-Jo-1 antibody positivity		P	
	Heliotrope rash	Creatine kinase			N	(97)
DM subtype	/	/	Anti-TIF1γ, Anti-SAE, and Anti-Mi2 antibodies positivity	/	N	(100)
Rapid onset of DM		Elevated ESR; Creatine kinase	/	/	N	(101)
	Raynaud phenomeno				P	
Rapid onset of DM	Periungual erythema; Cutaneous necrosis or ulceration	/	/	/	N	(102)
Refractory or drug-resistant DM		/	/	Elevated levels of CA-125	N	(103)
	Arthritis or arthralgia				P	
Absence of ILD	Dysphagia	/	/	/	N	(104)
DM complicated by obesity	/	/	/	/	N	(105)
/	Arthritis or arthralgia	/	Anti-Jo-1 antibody positivity	/	P	(106)
/	Periungual erythema	/	/	/	N	(107)
/	Cutaneous vasculitis	/	/	/	N	(108, 109)
/	Cutaneous necrosis or ulceration	Creatine kinase	/	/	N	(110)
/	Heliotrope rash	/	/	/	N	(111)
/	/	Elevated ESR		/	N	(112)
/	/	/	Anti-TIF1γ antibodies positivity	/	N	(113, 115)
			Anti-Jo-1 antibody positivity		P	
/	/	/	Anti-TIF1γ and Anti-SAE antibodies positivity	/	N	(114)
/	/	/	Anti-NXP2 antibodies positivity	/	N	(117)
/	/	/	Anti-SAE antibodies positivity	/	N	(119)
/	/	/	ANA positivity	/	P	(120)
/	/	/	Anti-Mi2 antibodies positivity	/	N	(121)
/	/	/	/	Elevated levels of CA-125 and CA19-9	N	(122)
/	/	/	/	Elevated levels of CA-125	N	(123)

P, Positive; N, Negative; DM, Dermatomyositis; ILD, Interstitial lung disease; ESR, Erythrocyte sedimentation rate; CRP, C-reactive protein; TIF-1γ, Transcription Intermediary Factor 1 Gamma; NXP2, Nuclear Matrix Protein 2; SAE, Small Ubiquitin-like Modifier Activating Enzyme; ANA, Antinuclear antibodies; CA-125, Cancer Antigen 125; CA19-9, Carbohydrate Antigen 19-9.

### 3.1 Demographic factors

Age: Malignancies are more common in DM patients aged 40 years or above (58, 71, 97). Several meta-analyses of relevant studies have validated older age as a risk factor of malignancies in DM patients (34, 98, 99).

Gender: In several studies, outcomes from multivariate analyses have supported that male gender is correlated with a greater risk of malignancies (10, 52, 63). Besides, several meta-analyses of relevant studies have validated male gender as a risk factor of malignancies in DM patients (34, 98, 99).

### 3.2 Clinical features

Disease subtype: Among the subtypes of IMM, patients with DM have a higher chance of malignancies compared to other subtypes, and the risk of cancer in IIM is concentrated among patients with DM (19, 20, 100). A recent meta-analysis further confirmed the DM subtype as a risk factor for malignancies (34).

Rapid onset of DM: Several studies showed that a rapid onset of DM had predictive value for the presence of malignancies in DM patients (101, 102). Outcomes from multivariate analyses also supported rapid onset of skin as a risk factor of malignancies in DM patients (102). A meta-analysis of relevant studies validated rapid onset of myositis as a risk factor of malignancies in DM patients (99).

Refractory to therapy: Several studies showed that refractory or resistant to therapy was correlated with a greater risk of malignancies in DM patients (95, 103).

Interstitial lung disease (ILD): In several studies, outcomes from multivariate analyses have supported that the absence of ILD is correlated with a greater risk of malignancies (10, 63, 66, 67, 104). Meta-analyses of relevant studies have validated ILD as a protective factor against malignancies in DM patients (34, 98, 99).

Comorbidities: One study reported that diabetes was correlated with a greater risk of malignancies in DM patients (10). Another study by Allenzara et al. revealed that obesity was a risk factor for cancer development in DM patients (105).

### 3.3 Symptoms and signs

Arthritis or arthralgia: Some studies have revealed that arthritis was correlated with decreased risk of malignancies among DM patients (10, 67, 80, 103, 106). Several meta-analyses of relevant studies validated arthritis as a protective factor of malignancies in DM patients (98, 99).

Fever: Some studies revealed that fever was correlated with decreased risk of malignancies among DM patients, suggesting fever as a protective factor of malignancies in DM patients (10, 67, 80).

Raynaud phenomenon: Some studies revealed that Raynaud phenomenon was correlated with decreased risk of malignancies among DM patients (10, 20, 80, 101). Several meta-analyses of relevant studies validated Raynaud phenomenon as a protective factor of malignancies in DM patients (34, 99).

Dysphagia: Many studies revealed that dysphagia was correlated with risk of malignancies among DM patients (10, 63, 66, 67, 104). In several studies, outcomes from multivariate analyses supported that dysphagia was correlated with greater risk of malignancies in DM patients (63, 66, 104). Several meta-analyses of relevant studies have further validated dysphagia as a risk factor of malignancies in DM patients (34, 98, 99).

Periungual erythema: Some studies have revealed that periungual erythema was correlated with risk of malignancies among DM patients (102, 107). Outcomes from multivariate analyses supported that periungual erythema was correlated with risk of malignancies (102).

Cutaneous vasculitis: Several studies reported that vasculitis in skin biopsies had a predictive value for the presence of malignancies in DM patients (108, 109). Meta-analyses of relevant studies validated cutaneous vasculitis as a risk factor of malignancies in DM patients (99).

Cutaneous necrosis or ulceration: Several studies reported that cutaneous necrosis or ulceration has a predictive value for the presence of malignancies in DM patients (80, 102, 110). Outcomes from multivariate analyses supported that cutaneous necrosis or ulceration was correlated with greater risk of malignancies (102). Several meta-analyses of relevant studies validated cutaneous necrosis as an important risk factor of malignancies in DM patients (34, 98, 99).

Heliotrope rash: Several studies reported that heliotrope rash has a predictive value for the presence of malignancies in DM patients (97, 111). Outcomes from multivariate analyses also supported that heliotrope rash was correlated with risk of malignancies (97).

### 3.4 Laboratory parameters

Erythrocyte sedimentation rate (ESR): Some studies revealed that elevated ESR was correlated with risk of malignancies among DM patients (101, 112). Outcomes from multivariate analyses supported that elevated ESR was correlated with risk of malignancies (63). Meta-analyses of relevant studies validated elevated ESR as an important risk factor of malignancies in DM patients (99).

Creatine kinase: Some studies revealed that elevated creatine kinase was correlated with risk of malignancies among DM patients (101, 110). However, other studies revealed that elevated creatine kinase was not correlated with greater risk of malignancies among DM patients (97). Several meta-analyses of relevant studies validated elevated creatine kinase as a risk factor of malignancies in DM patients (34, 99).

C-reactive protein (CRP): A meta-analysis showed elevated CRP as an important risk factor of malignancies in DM patients (99).

Albumin: A study by Chang et al. revealed that decreased albumin was correlated with greater risk of malignancies in DM patients (67).

### 3.5 Autoantibodies

#### 3.5.1 Myositis-specific autoantibodies

Anti-TIF1γ antibodies: Anti-TIF-1γ antibodies, previously named as anti-p155 antibodies, have been identified as myositis-specific autoantibodies and represent a significant and major risk factor for cancer-associated dermatomyositis (83, 113–115). Anti-TIF1γ antibodies have a high predictive value for malignancies in DM patients, and its utility in routine clinical practice has been validated by many studies (52, 100). Another systematic review and meta-analysis of 18 studies with a total of 1,962 DM patients further confirmed that anti-TIF-1γ antibodies were a valuable tool to identify DM patients with a higher risk of cancer (116). Apart from anti-TIF1γ antibodies, two other subtypes of anti-TIF1 antibodies, including anti-TIF-1α antibodies and anti-TIF-1β antibodies, have been identified (83). However, neither anti-TIF-1α antibodies or anti-TIF-1β antibodies are biomarkers for malignancy in DM patients. Anti-TIF-1γ antibody coexisting with other myositis-specific autoantibodies may increase the risk of cancer among DM patients, such as anti-NXP2 antibody.

Anti-NXP2 antibodies: Anti-NXP2 antibodies are myositis-specific autoantibodies and are frequently found in DM patients (52, 117). Some studies found that anti-NXP2 antibodies were correlated with risk of malignancy in DM patients (114, 117). Outcomes from multivariate analyses supported that anti-NXP2 antibodies were correlated with greater risk of malignancies in DM patients (52). However, two meta-analyses of relevant studies in IIM patients did not find a significant difference in the incidence of anti-NXP2 antibody between those IIM patients with and without cancer (34, 118). Moreover, it has been well-documented that DM patients with both Anti-NXP2 and Anti-TIF1γ positive antibodies had more increased risk of cancer (52). Therefore, it is of great importance to carry out systemic cancer screening among these DM patients, particularly in the first three years after DM diagnosis.

Anti-SAE antibodies: Findings from several studies found that anti-SAE antibodies were correlated with a lower increased risk of cancer in DM patients (100, 114, 119). Outcomes from multivariate analyses also supported that anti-SAE antibodies were correlated with elevated risk of malignancies in DM patients (119). However, DM patients positive with both anti-SAE and Anti-TIF1γ antibodies might have much more increased risk of cancer. This finding still needs to be validated in future studies.

Anti-Jo-1 antibody: In several studies, DM patients with malignancies less commonly exhibited anti-Jo-1 antibody compared with those without malignancies, and anti-Jo1 antibody was a protective factor of malignancies in DM patients (95, 106, 113). Two meta-analyses of relevant studies validated anti-Jo-1 antibody as a protective risk factor of malignancies in DM patients (34, 99). This finding still needs to be validated in future studies.

Anti-Mi2 antibodies: Several studies found that anti-Mi2 antibodies were correlated with risk of malignancy in DM patients (100, 121). Besides, DM patients positive with both anti-Mi2 and anti-TIF1γ antibodies might have higher risk of malignancies, which still needs to be validated in more studies.

#### 3.5.2 Myositis associated autoantibodies

Antinuclear antibodies (ANA) positivity: Some studies found that ANA positivity was correlated with lower incidence of malignancy in dermatomyositis (80, 120). Outcomes from multivariate analyses supported that ANA positivity was correlated with lower risk of malignancies in DM patients (120). This finding needs to be validated in more studies. Little is known about the association of other myositis associated autoantibodies with cancer risk among DM patients, such as anti-PM-Scl, anti-RO60, anti-RO52, anti-U1RNP, anti-Ku, anti-cN-1A, and anti-La antibodies. More future studies are warranted for further investigation. Whether the coexistence of myositis associated autoantibodies and anti-TIF1γ or other myositis-specific autoantibodies could increase the risk of cancer remains largely unknown.

### 3.6 Serum cancer markers

Serum cancer markers such as carcinoembryonic antigen (CEA), alpha-fetoprotein (AFP) and carbohydrate antigen 125 (CA125) are common and useful markers for assessing the risk of tumors. Their roles in assessing cancer risk in DM patients have been explored in several studies. For instance, several studies revealed that elevation of CA125 was correlated with an increased risk of solid cancers in DM patients (103, 122). Similarly, elevation of CA19–9 was found to be an increased risk of solid cancers in DM patients (122). In terms of ovarian cancer screening in DM patients, a study involving 14 female DM patients showed that elevated CA-125 had a sensitivity of 50% and a specificity of 100% for detecting early ovarian cancer in DM patients, highlighting its potential utility (123).

## 4 Cancer screening in DM patients

There is still a lack of in-depth research in cancer screening in DM patients, and the evidence for recommendations in cancer screening guideline in DM patients is limited (31, 95). This section reviews current methods used for cancer screening in DM patients and summarizes key findings from related research.

### 4.1 The value and challenges of malignancy evaluation in DM patients

DM patients have an increased risk of malignancies, underscoring the critical importance of early detection and treatment (124). The significance of early detection of malignancies in improving survival rates and quality of life of cancer patients has long been established (29, 30). Though no study has yet determined whether earlier diagnosis of malignancy improves outcomes in DM patients, there is no doubt regarding the significance of early detection of malignancies in DM patients. However, malignancy screening in DM patients poses significant challenges.

Conventional cancer screening methods like imaging examinations and tumor marker tests have some limitations caused by factors such as medical costs, potential side effects from imaging, and low patient compliance with screening (125). In a study by Sparsa et al., routine initial screenings failed to detect four malignancies, three of which were later found at advanced stages through more extensive evaluation (101). Cox NH et al. reported that routine cancer screening did not increase the detection of malignancies in DM patients suspected clinically or from abnormal simple investigations (126). A cross-sectional study suggested that physical examinations and imaging techniques often failed to detect early ovarian cancer in DM patients, with many ovarian malignancies being diagnosed at advanced stages (127). Those findings above suggest that conventional cancer screening methods are not effective in detecting malignancies among DM patients. Further research is needed to address these challenges and develop novel, effective cancer screening techniques.

Cancer sites with elevated risks in DM patients are not typically covered by conventional screening guidelines from the U.S. Preventive Services Task Force (USPSTF) or the American Cancer Society (ACS). Callen JP’s study of 57 DM patients with malignancies found that most (>90%) tumors occurred in areas not routinely screened, undermining the utility of standard malignancy evaluations (128). Kundrick et al. similarly concluded that age- and sex-appropriate cancer screening methods recommended by USPSTF and ACS guidelines might miss the majority of occult malignancies in young DM patients (129). Therefore, an optimized, evidence-based malignancy screening approach tailored to the characteristics of DM patients is needed, which may facilitate early detection and improve outcomes in DM-associated cancers. To enhance the efficacy of cancer screening, DM patients should undergo personalized cancer screening tailored to their specific risks, encompassing the cancer types most prevalent in DM (32).

### 4.2 Cancer screening tools

Common tools for cancer screening in DM patients mainly include physical examinations, laboratory tests, imaging examinations, and tissue biopsies (**Table 3**). Clinicians should integrate these methods based on patient clinical presentations and features to increase early detection of potential malignancies. When selecting cancer screening methods, factors such as accuracy, feasibility, and cost-effectiveness should be considered.

**Table 3:** Conventional cancer screening tools or strategies.

Tools	Screening items	Advantages	Disadvantages	Ref.
Physical examinations	Lumps, skin changes, enlarged lymph nodes, etc.	Direct observation of abnormal manifestations	Cannot confirm diagnosis, further testing required	(34, 63, 66)
Laboratory tests	Specific biomarkers associated with malignancies	Abnormal values may indicate certain malignancies	Cannot confirm diagnosis, further testing required	(34, 63, 67, 99)
X-ray examination	Breasts and lungs	Cost-effective and accessible screening options	Limited in detecting minor lesions or soft tissue tumors	(130)
CT or LDCT	Whole body	Facilitates early tumor detection, precise localization and staging, guides biopsy	Radiation exposure; possible false positives in patients with myositis	(131–133)
MRI	Whole body	Visualizes soft tissues without radiation; provides detailed information about tumor size and local spread	Expensive, limited availability in some settings	(134–138)
PET-CT	Whole body	Offers detailed info on metabolic activity and anatomical structures; detects early and advanced tumors; can evaluate muscle inflammation; single PET-CT test can be comparable to multiple combined screenings; high rate of confirmed diagnosis of screened suspected cases	Limitations such as cost, availability and exposure to ionising radiation; the literature does not support that screening is more effective	(32, 139–145)
Ultrasound examination	Breasts, thyroid, liver, kidneys, reproductive organs, etc.	Non-invasive; visualizes internal structures; helpful in assessing muscle inflammation	Initial screening tool; further confirmation needed	(146, 147)
Endoscopic examination	Gastrointestinal, respiratory or other hollow organs	Direct visualization and sampling; accurate diagnosis and staging of certain tumors	Lower coverage of cancer types	(148)
Tissue biopsies and cytology	Suspected tumor sites	Provides histological, grading, and molecular characteristics information	Invasive	(45)

CT, Computed tomography; LDCT, Low-dose computed tomography; MRI, Magnetic resonance imaging; PET-CT, Positron emission tomography/computed tomography; USPSTF, The U.S. Preventive Services Task Force; ACS, The American Cancer Society; DM, Dermatomyositis.

Physical examinations: Physical examinations are essential in cancer screening for detecting visible abnormalities and assessing overall health. Physicians should carefully evaluate patients for signs such as lumps, skin changes, or enlarged lymph nodes, which may indicate risk of malignancies.

Laboratory tests: Laboratory tests play a crucial role in cancer screening by assessing specific biomarkers associated with malignancies. For instance, cancer-associated autoantibodies such as anti-TIF-1γ antibodies and common serum cancer markers such as CA-125 are necessary laboratory tests in the malignancy evaluation of DM patients.

X-ray examination: X-ray examinations such as mammogram and chest radiography, are pivotal in cancer screening (130). Mammogram is essential for detecting breast cancer in its early stages by visualizing microcalcifications and masses, while chest radiography can detect lung tumors, nodules, or other pulmonary abnormalities. Despite limitations in detecting small lesions or soft tissue tumors compared to advanced imaging, X-rays offer cost-effective and accessible screening options for breast cancer and lung cancer in DM patients.

Computed tomography (CT) imaging: CT imaging is pivotal in cancer screening due to its ability to produce detailed cross-sectional images of the body. It can facilitate early detection, precise localization, and staging of tumors across various anatomical sites, including the chest, abdomen, and pelvis (131). Despite its advantages in imaging solid tumors and guiding biopsies, CT can cause ionizing radiation exposure, limiting their use in repeated screenings. Low-dose CT (LDCT) may be considered specifically for lung cancer screening in DM patients, with baseline scans of the chest, abdomen, and pelvis also being potential options (132). CT imaging of the chest, abdomen, and pelvis remains a viable screening option for DM patients at high risk of malignancies. A study involving IIM patient showed that CT scans of the chest, abdomen, and pelvis have a high diagnostic yield but can also yield false positive results for concurrent cancers in IIM (133). However, given that DM patients are susceptible to multiple types of cancer, this method may ignore some common malignancies correlated with DM. Further studies assessing the accuracy and cost-effectiveness of CT scans in detecting malignancies among DM patients are needed.

Magnetic resonance imaging (MRI): MRI plays a crucial role in cancer screening due to its high-resolution images and ability to visualize soft tissues in multiple planes without ionizing radiation. It can provide detailed information about tumor size, extent, and involvement of adjacent structures. MRI can provide much support and lead to more efficient screening of common solid tumors especially prostate cancer and breast cancer (134). Whole-body magnetic resonance imaging (WB-MRI) is currently recommended for cancer screening in adults and pediatric subjects with certain cancer predisposition syndromes (135, 136). Despite its advantages, MRI may be limited by medical cost and availability. Through several studies including case reports found that MRI may provide help in identifying cancers in DM patients (137, 138), the evidence for the use of MRI in cancer screening in DM patients is still lacking, and further studies proving the importance of MRI in detecting cancers among DM patients are needed.

Positron emission tomography/computed tomography (PET-CT): PET-CT is a powerful imaging method used in cancer screening especially for those possible cancer signs or signals have been detected by other screening methods (139). PET-CT can provide detailed images of metabolic activity and anatomical structures within the body, and is particularly valuable for detecting and staging various cancers. Despite its advantages, PET-CT has limitations such as cost, availability, and exposure to ionizing radiation. PET-CT can simultaneously evaluate muscle inflammation and detecting malignancies of DM patients, and several studies have explored the use of PET-CT in cancer screening among DM patients (140, 141). A study by Selva-O’Callaghan et al. showed that a single PET/CT examination for diagnosing malignancies was comparable to conventional screening, which includes multiple tests such as thoracoabdominal CT, mammography, gynecologic examination, ultrasonography, and tumor marker analysis (142). Another study by Li et al. screened 65 DM patients with PET/CT, and 19 patients were suspected to have malignancies, with 17 confirmed to have malignant tumors by biopsy (143). In a study by Trallero-Araguás et al., 77 patients underwent PET/CT for occult cancer screening, and the area under the curve (AUC) for PET/CT in diagnosing CAM in patients with myositis at disease onset was 0.87 (95% CI 0.73-0.97) (32). A study by Kundrick et al. showed that positron emission tomography (PET) including PET-CT costed less to patients than conventional cancer screening methods in DM patients (144). However, a study by Maliha et al. showed that PET/CT did not detect any malignancies that were detected by conventional cancer screening tests and resulted in more additional biopsies than conventional screening, which does not support PET/CT as a useful cancer screening tool for IIM patients compared to conventional methods (145). Therefore, further studies are needed to evaluate the role of PET/CT in the management of DM patients (139).

Ultrasound examination: Ultrasound examination, or ultrasonography, plays a pivotal role in cancer screening due to its non-invasive nature and ability to visualize internal structures with high resolution. It is widely used to detect tumors in various organs such as the breast, thyroid, liver, kidneys, and reproductive organs. In cancer screening, ultrasound serves as an initial diagnostic tool for detecting abnormalities that may require further evaluation through biopsy or additional imaging modalities like CT or MRI. The role of ultrasound examination in evaluating muscle inflammation has been explored by some studies, but its use in cancer screening among DM patients has not been evaluated (146, 147).

Endoscopic examination: Endoscopic examinations play a crucial role in cancer screening by providing direct visualization and biopsy of suspicious lesions within the gastrointestinal tract, respiratory tract, or other internal organs. Endoscopy allows for tissue biopsy for histopathological examination, aiding in the accurate diagnosis of malignancies and determination of tumor stage and grade. Hower, endoscopic examinations such as upper or lower gastrointestinal endoscopy, and nasoendoscopy have low coverage of cancers occurring in DM patients, and cannot be recommended for all DM patients. A study by Kidambi et al. found that endoscopic examinations such as upper endoscopy and colonoscopy had low yield in the identification of gastrointestinal cancers in DM patients (148). Therefore, endoscopic examinations need to be recommended for those DM patients who have evidence for high risk of certain types of cancers.

Tissue biopsies and cytology: Tissue biopsies are cornerstone diagnostic tools in cancer screening, providing essential information about tumor histology, grade, and molecular characteristics. Cytology, on the other hand, involves the examination of cells shed or aspirated from tumors or their surrounding tissues, typically for cancers located in accessible sites like the cervix (Pap smear) or lungs (sputum cytology). Despite their invasive nature, tissue biopsies and cytology remain indispensable in cancer management for all patients including DM cases, which can provide critical insights into disease progression and facilitating personalized treatment tailored to individual patient’ molecular profiles and characteristics.

### 4.3 Promising novel cancer detection tools for cancer screening in DM patients

Conventional cancer screening tools such as imaging examinations and tumor marker tests are not cost-effective or effective in detecting malignancies among DM patients, suggesting the unmet need in developing novel effective cancer detection tools. Advances in molecular sciences and high throughput screening have facilitated the development of innovative screening tests for cancer (**Table 4**), such a DNA methylation-based cancer screening tests (149). For instance, by detecting genetic mutations and biomarkers correlated with tumors in circulating tumor DNA (ctDNA), cell-free DNA (cfDNA) or circulating tumor cells (CTCs), liquid biopsies are emerging as powerful tools in cancer screening (150). This approach enables early detection of cancers, even before clinical symptoms manifest, which holds remarkable promise for revolutionizing cancer detection and is crucial for improving treatment outcomes. As technology advances and more studies confirm their efficacy, the utility of those liquid biopsies is increasingly and can be used as at least a complement to existing cancer screening tests (151). Among those tests, multi-cancer early detection test (MCED) can detect cancer signals from cfDNA or ctDNA, and can simultaneously detect multiple cancers and is promising to revolutionize cancer screening (152). Several prospective cohort studies support the feasibility of MCED for cancer screening, but also underscore the need for further research to increase its accuracy and reduce false-positive results (153, 154).

**Table 4:** Novel cancer screening tools of DM and international guideline for IIM-associated cancer screening.

Category		Advantages	Ref.
Tools	Cancer screening based on DNA methylation; liquid biopsy tests: ctDNA, cfDNA or CTCs detection; MCED	Enables early cancer detection, even before clinical symptoms appear; allows simultaneous detection of multiple cancers	(149–154)
	Anti-BNLF2	Novel biomarker for nasopharyngeal cancer screening; higher sensitivity, specificity, and positive predictive value	(155)
	Cancer risk prediction models	Helps establish a standardized cancer screening workflow; improves screening efficiency; integrates multiple clinical parameters; enables accurate prediction and individualized risk-based screening	(103)
Guideline	Recommendations and consensus for IIM-related cancer screening	Recommends the timing and frequency of cancer screening based on each patient’s risk profile	(157)

DM, Dermatomyositis; IIM, Idiopathic inflammatory myopathy; ctDNA, Circulating tumor DNA; cfDNA, Cell-free DNA; CTCs, Circulating tumor cells; MCED, Multi-cancer early detection test; Anti-BNLF2, Anti-Epstein-Barr Virus BNLF2b immunoglobulin G antibody.

Some novel but effective tests have been developed for specific types of cancers. Anti-Epstein-Barr Virus BNLF2b immunoglobulin G (IgG) antibody (Anti-BNLF2) has developed as a promising novel biomarker for nasopharyngeal carcinoma screening test with higher sensitivity, specificity, and positive predictive value than standard methods (155).

Apart from those promising cancer detection tests, cancer risk prediction models may help to establish a more optimized cancer screening workflow and further improve the efficacy of cancer screening. Cancer risk prediction models can integrate the predictive effects of multiple clinical parameters associated with cancer risk in DM, and may have a good predictive ability in evaluating cancer risk in DM patients, thus promoting a more individualized risk-based cancer screening. A study by Wang et al. developed a cancer risk prediction model by integrating male gender, glucocorticoid therapy resistance, older age, elevated CA125, positive anti-TIF1-γ antibody, arthralgia and elevated lymphocyte count, and the AUC of this model in predicting malignancy in PM/DM patients was 0.89 (95%CI: 0.85-0.92), with a sensitivity of 78% and a specificity of 86% (103). Further studies using large number of participants and prospective design are recommended to develop cancer risk prediction models with both high sensitivity and high specificity.

### 4.4 International guideline for idiopathic inflammatory myopathy-associated cancer screening

Although age- and gender-appropriate cancer screening routinely recommended for the general population can be applied among DM patients, it is not appropriate especially for those with significantly elevated risk of malignancies (31, 156). A risk stratification-based cancer screening strategy can lead to a more individualized approach for DM patients compared to recommendations for the general population (31, 156). To promote cancer screening and improve outcomes, evidence- and consensus-based recommendations for IIM-associated cancer screening were developed by International Myositis Assessment and Clinical Studies Group (IMACS) (**Table 4**) (157). The International Guideline for Idiopathic Inflammatory Myopathy-Associated Cancer Screening developed by IMACS stratifies IIM patients into standard, moderate, or high risk based on IIM subtype, autoantibody status, and clinical features. Recommended cancer screening methods by IMACS include a basic panel comprising chest radiography and preliminary laboratory tests, and an enhanced panel including CT and tumor markers (157). The timing and frequency of cancer screening via basic and enhanced panels are further recommended according to each patient’s risk status (157).

Real-world implications of the IMACS cancer screening guideline for IIM patients show that its implementation could significantly impact clinical practice and potentially result in substantial additional economic burden. However, guideline non-compliance could occur due to the lack of repeated annual screening in the three years post-diagnosis for high-risk individuals as outlined in this guideline (158). The effect of the IMACS cancer screening guideline on the early detection of malignancies among DM patients still needs to be explored in future studies.

## 5 Conclusions and future perspectives

There is substantial evidence supporting significantly elevated incidence of malignancies in DM patients. DM patients, particularly those within three years of onset, face heightened cancer risk and should undergo thorough and systematic cancer screening tailored to their individual risk stratification. Clinicians should carefully assess malignancy risk during DM diagnosis and treatment, conducting regular cancer screening and monitoring to facilitate early detection and intervention. Conventional cancer screening tools such as imaging examinations and tumor marker tests are suboptimal due to medical burden, side effects, and low compliance rates. Current cancer screening workflows available for DM patients largely mirror those used in the general population but may not fully address DM-specific characteristics.

Several unresolved issues on malignancies among DM patients require further researches. Firstly, the incompletely understood pathophysiology of DM-associated cancers has posed challenges for both precise diagnosis and effective treatment of malignancies among DM patients. To promote early detection and management of DM-associated cancers, deeper exploration into the precise mechanisms underlying malignancy in DM patients, including interactions among immune system abnormalities, genetic factors, and environmental influences, is needed. Secondly, risk factors of malignancy in DM patients still need to be defined and a more precise risk stratification is needed. Large-scale multicenter studies are necessary to validate existing research and explore potential new risk factors. Further studies using large number of participants and prospective design are recommended to develop cancer risk prediction models with both high sensitivity and high specificity. Thirdly, there is unmet need in developing novel effective cancer detection tools, and more effective cancer screening tools such as with both high accuracy and feasibility are warranted. cfDNA methylation-based MCED is an emerging powerful tool for revolutionizing cancer screening in DM patients, but further researches are still needed to increase its accuracy and reduce false-positive results. Further studies are recommended to explore the accuracy and cost-effectiveness of MCED in DM patient. Finally, an optimized, evidence-based malignancy screening workflow tailored to the characteristics of DM patients is essential to facilitate early detection and improve outcomes in DM-associated cancers. Though a risk stratification-based cancer screening strategy has been proposed by IMACS cancer screening guideline for IIM patients, the benefit of applying this guideline in DM patients still need to be proved by future research. To improve screening efficacy and facilitate earlier detection of malignancies, more personalized and more efficient screening workflows based on the features of DM and individual patient’s risk factors are warranted. These efforts will enhance early detection of malignancies in DM patients, leading to improved survival and treatment outcomes.

## Glossary

DM: Dermatomyositis
ANA: Antinuclear antibodies
IIM: Idiopathic inflammatory myopathies
CEA: Carcinoembryonic antigen
PM: Polymyositis
AFP: Alpha-fetoprotein
IBM: Inclusion body myositis
CA125: Carbohydrate antigen 125
CAM: Cancer-associated myositis
USPSTF: U.S. Preventive Services Task Force
ICIs: Immune checkpoint inhibitors
ACS: American Cancer Society
anti-TIF1γ: Anti-transcription intermediary factor 1γ
CT: Computed tomography
ADM: Adult dermatomyositis
LDCT: Low-dose Computed tomography
JDM: Juvenile dermatomyositis
MRI: Magnetic resonance imaging
SIR: Standardized incidence ratio
WB-MRI: Whole-body magnetic resonance imaging
RR: Rate ratio
PET-CT: Positron emission tomography/computed tomography
PMS: Paraneoplastic myositis syndrome
AUC: Area under the curve
MSAs: Myositis-specific autoantibodies
ctDNA: Circulating tumor DNA
CAAs: Cancer-associated autoantibodies
cfDNA: Cell-free DNA
LOH: Loss of heterozygosity
CTCs: Circulating tumor cells
TRIM: Tripartite motif
MCED: Multi-cancer early detection test
irAEs: Immune-related adverse events
Anti-BNLF2: Anti-Epstein-Barr Virus BNLF2b IgG antibody
ILD: Interstitial lung disease
IgG: Immunoglobulin G
ESR: Erythrocyte sedimentation rate
IMACS: International Myositis Assessment and Clinical Studies Group
CRP: C-reactive protein
